# Lymphatic dissemination of HPV-positive oropharyngeal squamous cell carcinoma: underlying mechanisms and treatment innovations

**DOI:** 10.3389/fimmu.2025.1601572

**Published:** 2025-06-02

**Authors:** Xinyu Li, Hui Qiu, Qiuji Wu

**Affiliations:** ^1^ School of Medicine, Zhongnan Hospital of Wuhan University, Wuhan, China; ^2^ Department of Radiation and Medical Oncology, Zhongnan Hospital of Wuhan University, Wuhan, China; ^3^ Hubei Key Laboratory of Tumor Biological Behavior, Zhongnan Hospital of Wuhan University, Wuhan, China; ^4^ Hubei Provincial Clinical Research Center for Cancer, Zhongnan Hospital of Wuhan University, Wuhan, China

**Keywords:** oropharyngeal carcinoma, human papillomavirus, lymphatic metastasis, immune microenvironment, immunotherapy

## Abstract

Cancers, with its rising incidence strongly linked to human papillomavirus (HPV) infection, particularly HPV16. HPV-induced OPSCC (HPV-OPSCC) exhibits distinct biological behaviors, including a high propensity for early lymphatic metastasis, occurring in most of cases, often presenting as cystic lymph node changes. The rising incidence of HPV-positive OPSCC is associated with specific mechanisms, particularly the characteristic biological behaviors driven by the E6/E7 oncoproteins: E7 disrupts cell cycle control by degrading pRb protein, while E6 inhibits apoptotic pathways through ubiquitination-mediated degradation of p53. Despite advances in treatment, HPV-OPSCC poses unique challenges due to its complex tumor microenvironment and immune interactions. Tertiary lymphoid structures (TLS) within the tumor microenvironment play a critical role in modulating anti-tumor immunity, correlating with improved clinical outcomes. Recent advances in immunotherapy, such as immune checkpoint inhibitors and HPV-specific vaccines, have shown promise in enhancing patient survival. This review explores the mechanisms of HPV-driven carcinogenesis, the clinical and molecular features of lymphatic metastasis, and the emerging role of TLS and immunotherapeutic strategies in HPV-OPSCC. By analyzing existing evidence, this review seeks to clarify the distinct biological features of HPV-associated oropharyngeal squamous cell carcinoma (HPV-OPSCC) and guide the development of novel treatment strategies aimed at enhancing clinical outcomes for patients. (OPSCC)

## 1 Introduction

With current estimates indicating approximately 25% of HNSCC cases originating in the oropharynx, OPSCC nowadays represents a substantial disease burden characterized via continuously escalating incidence rates. Escalating incidence rates of HPV (notably HPV16) infection, recognized as a key etiological factor in oropharyngeal malignancy formation, constitute the major determinant of this upward trend (1). Despite advances in treatment, including surgery, radiotherapy, and chemotherapy, HPV-OPSCC presents unique challenges, particularly its propensity for early lymphatic metastasis, which occurs in most of cases and significantly influences on prognosis (2, 3). Lymphatic metastasis is a hallmark of HPV-OPSCC progression, often manifesting as cystic changes in neck lymph nodes and exhibiting distinct patterns of spread compared to HPV-negative OPSCC (2). Studies have found that the spatial proximity between CD8+ T cells and PD-L1+ macrophages is enhanced in HPV-positive tumors, suggesting that immune escape may promote the lymphatic metastasis of cancer cells, which is associated with the high rate of lymphatic metastasis in HPV-OPSCC (4). In the management of early-stage human papillomavirus-associated oropharyngeal squamous cell carcinoma (HPV-OPSCC) with postoperative radiotherapy (PORT), lymphovascular invasion (LVI) has been identified as an independent adverse prognostic factor, potentially linked to its role in promoting lymphatic metastasis (5). HPV genome integration into host DNA can activate nearby oncogenes. Viral integration also induces genomic instability. These effects collectively promote lymphatic metastasis (6). The tumor microenvironment, including tertiary lymphoid structures (TLS), plays a critical role in modulating immune responses and shaping lymphatic metastatic behaviors. TLS are ectopic lymphoid formations and have been associated with improved clinical outcomes in HPV-OPSCC due to their role in enhancing local anti-tumor immunity (7). Recent advances in immunotherapy, particularly immune checkpoint inhibitors and HPV-specific vaccines, have shown promise in improving outcomes for HPV-OPSCC patients (8). However, the association between HPV status and immunotherapy response has not been fully elucidated, and validated biomarkers are currently lacking. This review aims summarizes the mechanisms of lymphatic metastasis in HPV-OPSCC, and the emerging role of TLS and immunotherapeutic strategies in HPV-OPSCC. By systematically consolidating contemporary evidence, this synthesis not only deciphers the distinct molecular landscape of HPV-OPSCC, but also establishes foundational knowledge for refining precision medicine strategies in clinical oncology.

## 2 Mechanisms of HPV-induced OPSCC

### 2.1 HPV-driven cell transformation and oncogene activation in OPSCC

While the precise oncogenic pathways of HPV in OPSCC remain incompletely characterized, accumulating evidence from translational studies strongly implicates HPV16 as the predominant etiological agent in oropharyngeal carcinogenesis. The high-risk HPV E6 and E7 genes are pivotal drivers of cell transformation, especially in basal squamous epithelial cells. For HPV types 16 and 18, both E6 and E7 proteins play essential roles in preventing senescence in human primary keratinocytes, potentially activating oncogenes and inducing carcinogenic cell proliferation (9, 10). E6 forms a complex as E6-AP, binding to p53 and promoting its degradation, thereby accelerating cell division and malignant transformation (11). Meanwhile, the E7 oncoprotein interacts with the retinoblastoma protein (pRb), disrupting its association with E2F transcription factors and facilitating unregulated progression into the S-phase of the cell cycle (12). The limited oncogenic potential demonstrated by HPV16/18 E2-E4-E5 genomic segments, which becomes further attenuated upon viral genome integration, may mechanistically explain their predominant association with non-malignant epithelial transformations rather than invasive carcinomas (13).

Some researchers propose that the cis-activating effect of HPV DNA on adjacent host genes has been postulated by investigators as a potential mechanism driving oncogenic transformation processes (11, 14). Hu et al. found that the E6/E7 proteins regulate the activity of key enzymes in the aerobic glycolysis pathway by influencing the binding of IGF2BP2 to the MYC m6A site (overexpression of IGF2BP2 in E6/E7-knockout CC cells). Ultimately, this reduces the glycolytic flux, leading to a decrease in cancer cell proliferation (15). Besides, hu et al. suggest that oncogenic human papillomaviruses (HPVs) could generate cir-cRNAs, some of which encompass the E7 oncogene (circE7), which is N6-methyladenosine (m6A) modified, preferentially localized to the cytoplasm, associated with polysomes. CircE7 can be translated and produce E7 oncoprotein (16). Other studies have noted that the integration of HPV DNA into chromosomes is a significant inducer of p53 gene mutations and may also lead to its overexpression (17). Palefsky et al. discovered that HPV16 infection is a cause of cellular transformation, likely due to alterations in the p53 and retinoblastoma proteins(pRb) triggered by HPV E6 and E7 (18). The cumulative evidence establishes HPV E6/E7’s pivotal function in p53 and pRb pathway disruption, which initiates cellular transformation and oncogenic signaling cascades, thereby mechanistically underpinning OPSCC pathogenesis.

### 2.2 Synergistic effects of HPV and other carcinogenic factors

Although HPV alone can induce malignant cell transformation, its carcinogenicity is often enhanced by synergistic interactions with other factors (14, 17). Oral tissues, exposed to various physical, chemical, and microbial agents, are particularly susceptible to the combined effects of HPV with smoking, alcohol, trauma, fungal infections, or other viruses, contributing to oral malignancies (17). Animal models confirm HPV’s role as a cofactor in carcinogenesis, with smoking facilitating HPV invasion and colonization in oral tissues (19). Additionally, HPV, smoking, and alcohol collectively induce oral cancer, suggesting a synergistic interaction. HPV’s carcinogenicity may also be influenced by hormone levels and immune status. For instance, The observed elevation in HPV prevalence among gravid populations, combined with augmented cervical carcinogenesis risk in prolonged contraceptive users, implies endocrine-mediated potentiation of viral genome duplication and mitotic activity (17, 19). Elevated hormone levels may enhance viral DNA expression, favoring carcinogenesis. Furthermore, higher HPV infection rates and tumor incidence in immunocompromised individuals, such as kidney transplant and acquired immunodeficiency syndromes (AIDS) patients, indicate latent HPV reactivation under such conditions (19).

## 3 Characteristics, detection and treatment of lymphatic metastasis in HPV-OPSCC

HPV-OPSCC often lacks early symptoms, and the existence of precancerous lesions remains controversial, with no such lesions found in 4,095 healthy individuals (20). Clinical data show that the lymphatic metastasis rate of HPV-OPSCC can exceed 90%, significantly higher than that of HPV-negative OPSCC (3). Additionally, Two-thirds of patients present with a neck mass, indicating lymphatic metastasis at diagnosis, creating a diagnostic and therapeutic blind spot (21).

### 3.1 Characteristics of lymphatic metastasis in HPV-OPSCC

Compared with HPV-negative OPSCC, HPV-OPSCC typically presents with smaller primary lesions but is more likely to develop early lymphatic metastasis, often manifesting as cystic changes in metastatic neck lymph nodes (cystic metastases). HPV-OPSCC also exhibits a distinctive pattern of lymph node invasion: the involved lymph nodes tend to be large and prone to extracapsular spread (2). Although HPV-OPSCC and HPV-negative OPSCC differ substantially in terms of their lymphatic metastasis rate and time to metastasis, they share similar locations and numbers of invaded lymph nodes, suggesting a fundamentally similar route of lymphatic invasion (22). Given the different pathogenic mechanisms and biological behaviors of HPV-OPSCC and HPV-negative OPSCC, the 8th edition of the American Joint Committee on Cancer (AJCC) staging manual has established an independent TNM staging system for HPV-OPSCC (21).

### 3.2 Detection of lymphatic metastasis in HPV-OPSCC

Positron emission tomography-computed tomography (PET-CT) is currently one of the most employed clinical methods for detecting lymphatic metastasis in head and neck tumors (23). However, a clinical study by Snyder et al. reported a misdiagnosis rate of 36–43% in detecting lymphatic metastasis in 49 HPV**-**OPSCC cases using PET-CT, with challenges in accurately determining the number and size of affected lymph nodes (24). Some researchers have established predictive models based on clinical presentation and imaging results to evaluate the risk of lymphatic metastasis in HPV-OPSCC patients; however, these models require validation through prospective studies (25). Fine-needle aspiration (FNA) biopsy is also insufficient for accurate detection, as aspirates from cystic metastatic lymph nodes in HPV-OPSCC resemble fluid from benign cystic lesions, potentially leading to diagnostic omissions (26). High-risk HPV testing on FNA samples is recommended for patients presenting with unexplained neck masses to determine whether these lesions might represent HPV-OPSCC metastases (26). HPV-RNA *in situ* hybridization has shown promise, with an 88.9% concordance rate with p16 immunohistochemical staining in detecting HPV in neck FNA samples (27).

### 3.3 Treatment and prognosis of lymphatic metastasis in HPV-OPSCC

Although HPV-associated oropharyngeal squamous cell carcinoma (HPV-OPSCC) is categorized as a distinct disease entity in the AJCC 8th edition staging system, current clinical management remains largely similar to that for HPV-negative OPSCC because of limited high-quality clinical evidence justifying disease-specific therapeutic strategies. (28). Patients with early HPV-OPSCC can undergo surgical monotherapy; however, most patients present with lymphatic metastasis and extracapsular spread at diagnosis, necessitating combined surgery, radiotherapy, and chemotherapy (28). It is important to emphasize that patients with lymphatic metastasis have a poorer prognosis compared to those without nodal involvement, underscoring the need to further elucidate the mechanisms of lymphatic metastasis and develop targeted interventions. Despite early and high rates of lymphatic metastasis, the overall prognosis of HPV-OPSCC is markedly better than that of HPV-negative OPSCC. A recent clinical study found that the 3-year overall survival rate was significantly higher (93%) among HPV-OPSCC patients than among HPV-negative OPSCC patients with a smoking history (46.2%) (28). The number of invaded lymph nodes exerts minimal effect on overall survival in HPV-OPSCC, contrasting sharply with other head and neck malignancies (29). The distinct pathobiological profile of HPV-OPSCC necessitates urgent elucidation of molecular mediators governing its preferential lymphatic dissemination, a prerequisite for optimizing nodal disease management protocols.

## 4 Exploration of lymphatic metastatic mechanisms in HPV-OPSCC

Metastatic colonization of lymphatic systems is mechanistically governed by synergistic contributions from genomic instability, epithelial-mesenchymal transition, and chemokine-mediated navigation. Seeing that the marked differences in lymphatic metastasis between HPV-OPSCC and HPV-negative OPSCC, several studies have sought to elucidate the specific mechanisms underlying lymphatic metastasis in HPV-OPSCC.

### 4.1 HPV^+^ OPSCC and epithelial–mesenchymal transition

Despite HPV-OPSCC’s sensitivity to radiotherapy and chemotherapy, its high lymphatic metastasis incidence remains paradoxical. EMT is critical for lymphatic metastasis (30). HPV E6/E7 upregulates EMT transcription factors (Slug, Twist, ZEB1, ZEB2), enhancing tumor proliferation and invasiveness (31). Nevertheless, PRKCZ, an oncogenic factor, promotes HPV-OPSCC progression but is inhibited by HPV E6-induced hypermethylation, suppressing EMT (32). Recent studies indicate that miRNA 34a significantly inhibits tumor stem cell proliferation, invasiveness, and EMT in HNSCC, with reduced miRNA 34a levels correlating with increased tumor invasiveness (33). The oncogenic proteins E6 and E7 of HPV can also enhance the tendency of tumor cells to metastasize to lymph nodes by activating the pathways related to epithelial-mesenchymal transition (EMT), such as the Wnt/β-catenin pathway (33). Cancer stem cells (CSCs) are pivotal for EMT and tumor metastasis, with markers like ALDH1A1, CD44, CD98, BMI1, and OCT4. Gunduz et al. reported lower CD44 and CD98 expression in HPV+ OPSCC specimens (34), while another study found reduced BMI1 expression in HPV+ OPSCC (35). Zhang et al. found that HPV-OPSCC contains more CSCs than HPV-negative OPSCC, with HPV E6 degrading p53 or blocking its acetylation, increasing CSC numbers and tumor invasiveness (36). Hufbauer et al. showed that HPV E6/E7 mediates the transition from stationary to migratory CSCs by regulating CD44 and EpCAM, promoting lymphatic metastasis in HPV-OPSCC (37). It shows that the intricate interplay between cancer stem cell plasticity and microRNA-mediated regulatory networks in HPV-driven oncogenesis is compellingly demonstrated by these experimental observations.

### 4.2 HPV-OPSCC and the vascular endothelial growth factor family

The VEGF family, particularly VEGF-C, VEGF-D, and their receptor VEGFR-3, plays a critical role in lymphangiogenesis and lymphatic metastasis in various malignancies, including HNSCC (38). Elevated VEGF-C/D expression in HNSCC correlates with increased peritumoral lymphatic vessel density (LVD), lymphatic metastasis, and poor prognosis, with VEGF-C serving as a predictive marker for metastasis (39, 40). In HPV-related tumors, HPV16-E6 seems to induce VEGF expression independently of TP53 inactivation, using the SP1 transcription factor for E6-mediated induction of the VEGF promoter (41). However, Baruah et al. found that circulating VEGF levels in HPV-OPSCC were similar to healthy controls, with no significant difference in VEGF-D expression among HPV-OPSCC, HPV-negative OPSCC, and healthy groups (42). This low VEGF expression contrasts with the lymphatic invasiveness of HPV-OPSCC, raising questions about whether HPV-OPSCC forms specific lymphatic metastases via VEGF-C-mediated lymphangiogenesis (**Table 1**).

**Table 1:** Mechanisms of HPV-induced OPSCC and lymphatic metastasis.

Mechanism	Molecules/Pathways	Functional Role	Implications
HPV Integration	E6, E7, E1/E2 disruption	E6 degrades p53; E7 inactivates RB, promoting cell cycle progression and oncogenesis.	High-risk HPV integration correlates with poor prognosis and therapeutic resistance.
Oncogene Activation	c-myc, p53, pRb	HPV DNA integration activates c-myc and other oncogenes, enhancing cellular transformation.	Targeted therapies against c-myc and p53 pathways may improve outcomes.
Epithelial–Mesenchymal Transition (EMT)	ZEB1, Slug, Twist, miRNA 34a, Wnt/β-catenin	HPV E6/E7 induces EMT transcription factors, promoting tumor invasiveness and metastasis.	EMT markers (e.g., ZEB1) may serve as prognostic biomarkers for lymphatic metastasis.
Cancer Stem Cells (CSCs)	CD44, EpCAM, BMI1, OCT4	HPV E6/E7 promotes CSC transition (CD44high/EpCAMlow), driving lymphatic metastasis.	Targeting CSCs may reduce metastasis and improve therapeutic efficacy.
VEGF Family	VEGF-C, VEGF-D, VEGFR-3	VEGF-C/D promotes lymphangiogenesis and lymphatic metastasis.	Anti-VEGF therapies (e.g., bevacizumab) may inhibit lymphatic spread.
Tumor Hypoxic Microenvironment	HIF-1α, E-cadherin, matrix metalloproteinases (MMPs)	Hypoxia induces EMT and CSC maintenance, enhancing tumor invasiveness.	Hypoxia-targeting therapies (e.g., HIF-1α inhibitors) may reduce metastasis.

### 4.3 HPV-OPSCC and the tumor hypoxic microenvironment

A hypoxic tumor microenvironment leads to elevated levels of hypoxia-inducible factor (HIF), which promotes EMT and increased secretion of matrix metalloproteinases by inducing downstream target genes, thereby enhancing tumor invasiveness (43). HPV-associated oropharyngeal squamous cell carcinoma (HPV-OPSCC) tumor cells may demonstrate hypoxia-inducible factor (HIF)-mediated suppression of the activation of E-cadherin and concurrent EMT, promoting increased invasive potential. Under hypoxic conditions, CSCs can maintain stemness and self-renewal capacity under the stimulation of HIF-1, further enhancing tumor invasiveness (43). However, another study showed that the tumor microenvironment of HPV-OPSCC lacks significant hypoxia, with minimal HIF expression, but displays significantly increased neovascular density around the cancer cells (44). This finding suggests that HPV-OPSCC may possess a unique mechanism of hypoxia.

## 5 Relationship between HPV-induced OPSCC and the immune microenvironment

The relationship between HPV-driven oropharyngeal squamous cell carcinoma (OPSCC) and the tumor immune microenvironment has been extensively characterized in contemporary research. HPV-positive OPSCC exhibits significantly higher infiltration of CD8+ T cells within the tumor microenvironment compared to HPV-negative cases, correlating with improved patient prognosis (45). Furthermore, the activation of interferon signaling pathways, such as IFN-γ, in HPV-positive OPSCC enhances tumor immunogenicity, promoting antigen presentation and T cell activation (46). However, despite the heightened immune activity in HPV-positive OPSCC, tumor cells can evade immune surveillance through mechanisms such as upregulation of PD-L1 expression. A study revealed that PD-L1 expression is significantly higher in HPV-positive OPSCC, potentially mediated by the HPV oncoprotein, which contributes to immune evasion (47).

Additionally, the tumor microenvironment in HPV-positive OPSCC is characterized by increased infiltration of immunosuppressive cells, including tumor-associated macrophages (TAMs) and regulatory T cells (Tregs). These cells secrete cytokines such as IL-10 and TGF-β, which suppress effector T cell function and facilitate immune escape (32, 47). Although PD-1/PD-L1 inhibitors show efficacy in some patients, the presence of regulatory T cells (Tregs) can promote an immunosuppressive microenvironment that limits therapeutic effectiveness (48). Studies have shown that Tregs further exacerbate the formation of an immunosuppressive microenvironment by recruiting and activating other immunosuppressive cells, such as myeloid-derived suppressor cells (MDSCs) and M2 macrophages (49). Tregs suppress the activation and cytotoxic function of CD8+ T cells by secreting inhibitory cytokines and through direct cell-cell contact, thereby enabling tumor cells to evade immune-mediated elimination (50, 51). Due to the immunosuppressive function of Tregs, single-target immunotherapy is difficult to be effective. Therefore, some strategies that jointly target Tregs are needed. Studies have found that IDO1 inhibitors can regulate the development and activation of Treg cells and granulocyte-derived suppressor cells (MDSCs), and inhibit effector T cells and natural killer (NK) cells. IDO1 can also promote the neovascularization of tumors by regulating the production of interferon-gamma (IFN-γ) and interleukin-6 (IL-6) (52, 53). Therefore, the combined use of IDO1 inhibitor and the checkpoint inhibitor can enhance the efficacy of cancer immunotherapy (54). In summary, Tregs promote the formation of an immunosuppressive microenvironment in HPV-OPSCC through multiple mechanisms, thereby driving immune evasion and resistance to immunotherapy. Targeting Tregs represents a promising therapeutic strategy to overcome these challenges and achieve breakthroughs in HPV-OPSCC treatment.

HPV viral antigens induce CD161+ CTL subsets that co-express activation markers (e.g., IFN-γ) and exhaustion markers (e.g., PD-1, CTLA-4), forming an immunosuppressive microenvironment to facilitate lymphatic metastasis. Other research indicates that integrins on the surface of tumor-derived exosomes (TDEs), such as α6β4, specifically target lymphatic endothelial cells, thereby activating EMT and inhibit T - cell function and establishing a pre-metastatic microenvironment that directs tumor cells to colonize within the lymph nodes (55). Therefore, despite demonstrating a high degree of immunogenicity in HPV-positive oropharyngeal squamous cell carcinoma (OPSCC), the immunosuppressive processes inherent to the tumor microenvironment continue to present a major obstacle to successful immunotherapeutic interventions.

## 6 Debate of tertiary lymphoid structures in HPV-induced OPSCC

Tertiary lymphoid structures (TLS) are ectopic immune aggregates in HPV-induced oropharyngeal squamous cell carcinoma (HPV-OPSCC) that exhibit dual roles in tumor immunity. While TLS formation may correlate with improved prognosis by supporting cytotoxic lymphocyte activity, their functional dysfunction-driven by immunosuppressive mechanisms like regulatory T cell infiltration and cytokine dysregulation-can paradoxically promote immune evasion and immunotherapy resistance. We next examine these conflicting roles and explore therapeutic strategies targeting TLS reprogramming to enhance treatment efficacy in HPV-OPSCC.

### 6.1 Role of tertiary lymphoid structures in HPV-induced OPSCC

TLS form from lymphoid and stromal cell aggregation in non-secondary lymphoid organs under pathological conditions, including autoimmune diseases, infections, transplant rejection, and malignancies (56). Persistent inflammation upregulates chemokines (e.g., CXCL13, IL-7), attracting lymphoid tissue inducer (LTi) cells (e.g., Th17, B cells, M1 macrophages) to inflamed sites (57, 58). LTα1β2 on LTi cells interacts with LTβR and IL-17 receptors on stromal cells, inducing VEGF-C release, high endothelial venule (HEV) formation, and adhesion molecule expression (VCAM-1, ICAM-1) (59). Macrophages and endothelial cells secrete IL-36γ, further enhancing VCAM-1, ICAM-1, and chemokines (IL-8, CCL2, CCL20), promoting HEV recruitment of lymphocytes and TLS maturation (60).

The role of TLS in HPV-positive OPSCC has garnered increasing attention. A study found that the presence of TLS in HPV-positive OPSCC is associated with higher CD8+ T cell infiltration and improved clinical outcomes (61). Tertiary lymphoid structures (TLS), residing within the tumor microenvironment, represent ectopic lymphoid formations that actively promote localized anti-tumor immune activity. In HPV-positive OPSCC, B cells and T cells within TLS collaborate to generate HPV-specific antibodies and cytotoxic T cell (CTL) responses, thereby enhancing anti-tumor immunity. The formation and maintenance of TLS are closely linked to the expression of chemokines such as CXCL13 and CCL21. High CXCL13 expression is associated with TLS formation and function in HPV-positive OPSCC, providing a theoretical basis for targeting chemokine networks to enhance TLS activity (56). Additionally, follicular helper T cells (Tfh) within TLS play a critical role in B cell differentiation and antibody production, further amplifying anti-tumor immune responses (62). However, TLS functionality is not uniformly effective across all patients, as some exhibit dysfunctional TLS characterized by B cell exhaustion or T cell impairment. Therefore, it is meaningful to further explore and focus on elucidating the mechanisms underlying the dysfunction of tertiary lymphoid structures (TLS), and to develop targeted strategies to restore their anti-tumor activity.

### 6.2 The mechanism of TLS dysfunction and treatment strategies in HPV-induced OPSCC

Immune checkpoint blockade (ICB) targeting PD-1/PD-L1 is a prominent immunotherapy. It has demonstrated significant efficacy in treating OPSCC. However, the overall response rate to PD-1/PD-L1 blockade in OPSCC remains below 20% (63). Studies have revealed that CD20+ B cells predominantly localize within TLS (64). Moreover, the presence of CD20+ B cells is associated with a more favorable prognosis for patients with OPSCC (65). The induction of TLS formation enhances the response to PD-1 blockade treatment in HPV-OPSCC mouse models. Therefore, promoting TLS formation may improve the response rates of HPV-OPSCC patients to ICB therapy (66). A study found that by regulating chemokines such as CXCL13 or lymphoid tissue inducers like LTα/β, the maturation and function of TLS can be promoted, and the anti-tumor immunity can be enhanced (67). Another study found that the PD-1 inhibitor pembrolizumab is effective in enhancing the response to immunotherapy. It demonstrated clinically meaningful anti-tumour activity in recurrent or metastatic squamous cell carcinoma of the head and neck (68).

In HPV-OPSCC, the maturity of TLS is closely linked to their functional capacity. Immature TLS may fail to effectively facilitate antigen presentation and lymphocyte activation, thereby compromising immunotherapy efficacy. In contrast, mature TLS are enriched with memory B cells, plasma cells, and CD4+ T cells, accompanied by upregulated expression of B-cell activation-related genes. Consequently, promoting TLS maturation could enhance the therapeutic response to immunotherapy in HPV-OPSCC (69).

**Figure 1:**
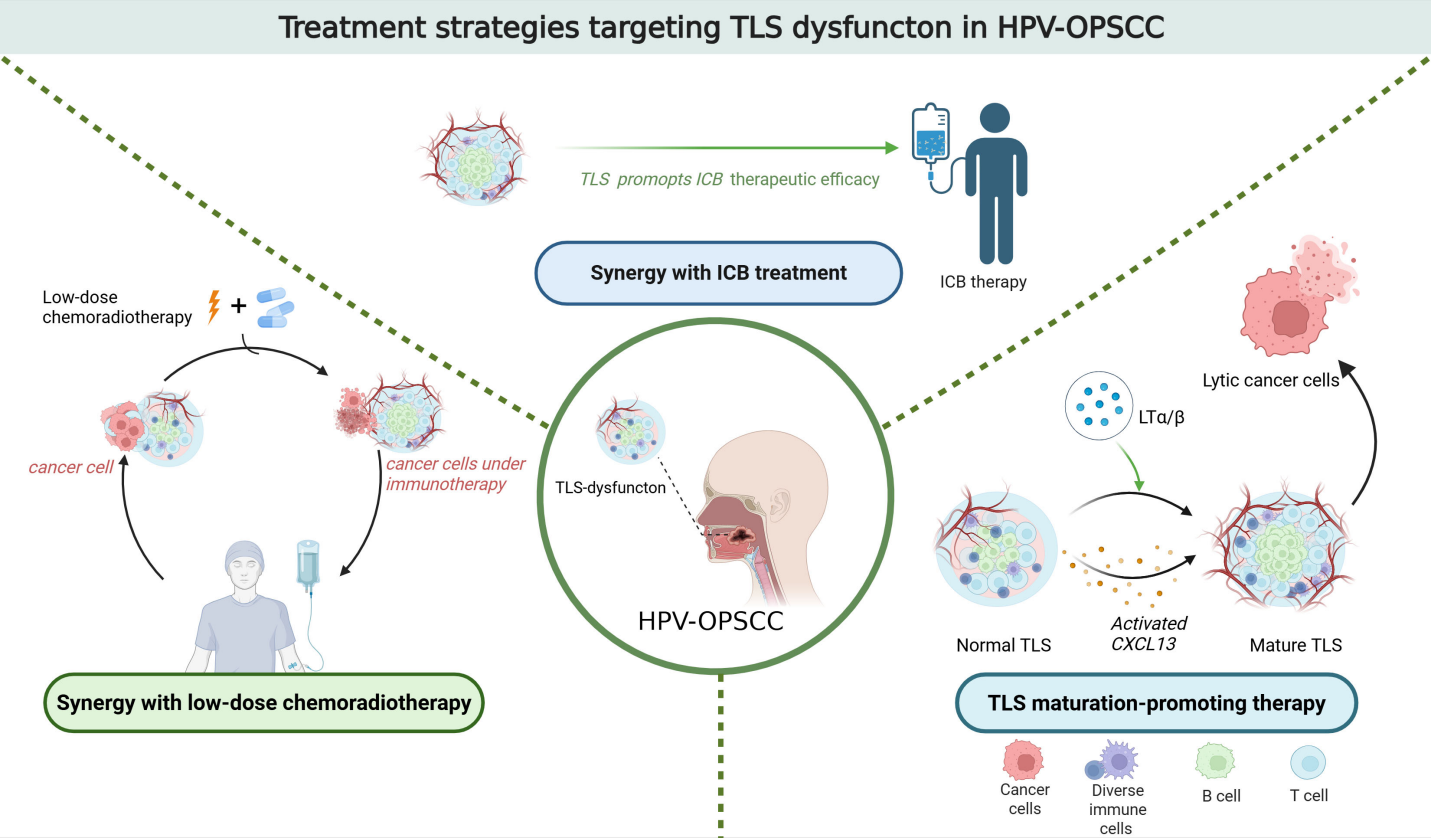
Treatment strategies targeting TLS dysfuncton in HPV-OPSCC.

Besides, in HPV-associated oropharyngeal squamous cell carcinoma (HPV-OPSCC), dysfunction of TLS may be linked to a significant reduction in CD8+ T cells and B lymphocytes within the tumor microenvironment. This association is particularly pronounced in cases of disease recurrence following chemotherapy or radiotherapy (70). Further research revealed T cell hat conventional chemoradiotherapy may disrupt TLS integrity, leading to deterioration of the local immune microenvironment. Consequently, this impairment reduces response rates to immunotherapy. Given favorable prognosis of HPV-OPSCC patients, decreasing therapeutic intensity-such as reducing radiation doses and avoiding chemotherapy, and minimizing overtreatment-could preserve TLS functionality and sustain antitumor immunity (71). A recent study found that neoadjuvant chemotherapy (NAC) can enhance the HPV-specific T cell response of the primary tumor and improve the survival rate of patients (72) (**Figure 1**).

## 7 Immunotherapeutic strategies for HPV-induced OPSCC

HPV-positive OPSCC shows stronger responses to immunotherapy, especially immune checkpoint inhibitors. The KEYNOTE-048 clinical trial reported that the PD-1 inhibitor pembrolizumab significantly improved overall survival in patients with HPV-positive OPSCC (61). Additionally, HPV vaccines have shown promise in treating HPV-associated OPSCC. A study in demonstrated that an HPV E6/E7 vaccine induced robust HPV-specific T cell responses and led to tumor regression in some patients (8). Despite these advancements, resistance to immunotherapy remains a significant challenge. Research identified that immunosuppressive cells, such as Tregs and myeloid-derived suppressor cells (MDSCs), within the tumor microenvironment can inhibit T cell function, contributing to immunotherapy resistance (73). To overcome these limitations, current research focuses on developing multimodal treatment approaches that combine immune checkpoint inhibitors with radiotherapy or chemotherapy to counteract therapeutic resistance and improve clinical outcomes. Furthermore, adoptive T cell therapies, such as chimeric antigen receptor (CAR) T cell therapy and tumor-infiltrating lymphocyte (TIL) therapy, have shown potential in clinical trials. For instance, a study demonstrated that CAR-T cells targeting HPV E6/E7 exhibited potent anti-tumor activity in HPV-positive OPSCC (74). These emerging immunotherapeutic strategies offer new hope for patients with HPV-positive OPSCC, underscoring the need for continued research to optimize treatment outcomes.

## 8 Conclusion

HPV-positive oropharyngeal squamous cell carcinoma (HPV-OPSCC) represents a distinct clinical and molecular entity within head and neck cancers, characterized by a high propensity for early lymphatic metastasis and a unique tumor-immune microenvironment. The coordinated engagement of HPV-derived oncogenic factors, immune regulation, and lymphotropic metastatic processes serves to elucidate the pathobiological complexity inherent in virally mediated tumor development. While tertiary lymphoid structures (TLS) have emerged as critical modulators of anti-tumor immunity, their functional heterogeneity emphasizes the need for further mechanistic studies to harness their full therapeutic potential.

Recent advancements in immunotherapy, particularly immune checkpoint blockade and HPV-targeted vaccines, have reshaped the treatment landscape, demonstrating promising efficacy in HPV-OPSCC. However, immune evasion mechanisms, including regulatory T cell infiltration and PD-L1 upregulation, continue to pose significant challenges, necessitating the development of combination strategies to strengthen therapeutic responsiveness. Future investigations should prioritize the refinement of prognostic biomarkers, enhance the development of immunotherapeutic approaches, and systematically characterize the molecular mechanisms governing lymph node metastasis. By integrating these insights, the field can move toward more precise and durable treatment strategies, ultimately improving patient outcomes in HPV-OPSCC.
